# Reduced erythrocyte membrane polyunsaturated fatty acid levels indicate diminished treatment response in patients with multi- versus first-episode schizophrenia

**DOI:** 10.1038/s41537-022-00214-2

**Published:** 2022-02-25

**Authors:** Nana Li, Ping Yang, Mimi Tang, Yong Liu, Wenbin Guo, Bing Lang, Jianjian Wang, Haishan Wu, Hui Tang, Yan Yu, Xiangxin Wu, Cuirong Zeng, Ting Cao, Hualin Cai

**Affiliations:** 1 Department of Pharmacy, the Second Xiangya Hospital of Central South University, Changsha, Hunan Province China; 2 Institute of Clinical Pharmacy, Central South University, Changsha, Hunan Province China; 3 Department of Psychiatry, the Second People’s Hospital of Hunan Province, Changsha, Hunan Province China; 4 Department of Pharmacy, Xiangya Hospital of Central South University, Changsha, Hunan Province China; 5 Institute of Hospital Pharmacy, Xiangya Hospital of Central South University, Changsha, Hunan Province China; 6 Department of Psychiatry, The Second Xiangya Hospital of Central South University, Changsha, Hunan Province China; 7 National Clinical Research Center on Mental Disorders, Changsha, Hunan Province China; 8 Department of Psychiatry, Changsha Psychiatric Hospital, Changsha, Hunan Province China

**Keywords:** Biomarkers, Schizophrenia

## Abstract

Antipsychotic effects seem to decrease in relapsed schizophrenia patients and the underlying mechanisms remain to be elucidated. Based on the essential role of polyunsaturated fatty acids in brain function and the treatment of schizophrenia, we hypothesize that disordered fatty acid metabolism may contribute to treatment resistance in multi-episode patients. We analyzed the erythrocyte membrane fatty acids in 327 schizophrenia patients under various episodes (numbers of patients: first-episode drug naïve 89; 2–3 episodes 110; 4–6 episodes 80; over 6 episodes 48) and 159 age- and gender-matched healthy controls. Membrane fatty acid levels and PANSS scales were assessed at baseline of antipsychotic-free period and one-month of follow-up after treatment. Totally, both saturated and unsaturated fatty acids were reduced at baseline when compared to healthy controls. Subgroup analyses among different episodes indicated that in response to atypical antipsychotic treatment, the membrane fatty acids were only increased in patients within 3 episodes, and this therapeutic effects on omega-3 index were merely present in the first episode. Results of fatty acid ratios suggested that dysregulations of enzymes such as D6 desaturase, D5 desaturase, and elongases for polyunsaturated fatty acids in patients with multi-episode schizophrenia could account for the differences. Additionally, certain fatty acid level/ratio changes were positively correlated with symptom improvement. The alterations of C22:5n3 and omega-3 index, gender, and the number of episodes were significant risk factors correlated with treatment responsiveness. Using targeted metabolomic approach, we revealed the potential mechanisms underlying abnormal fatty acid metabolism responsible for reduced treatment response in patients with multi-episode schizophrenia.

## Introduction

Antipsychotic maintenance treatment is critically important for the prevention of relapse in schizophrenia^1^. Poor compliance in patients with schizophrenia results in a high risk of relapse^2^. Moreover, antipsychotic treatment response is reported to be diminished in the second episode compared with the first episode during the follow-up of the same group of patients^3^. The problem of antipsychotic treatment resistance in subsequent psychotic episodes has been discussed but the underlying mechanisms remain unclear^4^. It was suggested that the research of biomarkers will initiate precision medication in psychiatry that will eliminate “treatment resistance”^5^. It is hypothesized that dopamine supersensitivity evoked by long-term antipsychotic treatment is linked to the tolerance of antipsychotic drugs^6^. And dopaminergic neurotransmission interacts with membrane phospholipids in cognitive deficits of schizophrenia^7^. The n-3 fatty acid/dopamine hypothesis of schizophrenia also suggests that decreases in the n-3 fatty acid levels induce hypofunction of the prefrontal dopamine system via decreasing dopamine vesicles, concentration, and D2 receptors^8^. Furthermore, a lipidomic analysis has suggested that changes in specific lipid profiles are associated with poor or good response to atypical antipsychotic treatment in schizophrenia^9^. Thus, it is critical to explore the role of membrane phospholipids in the treatment resistance of patients with multi-episode schizophrenia.

The membrane hypothesis of schizophrenia revealed that abnormal phospholipid metabolism in both neuronal and erythrocyte membranes may be involved in the etiology of schizophrenia^10^. Polyunsaturated fatty acids (PUFAs) and their metabolites regulate several processes, such as neurotransmission, neuroinflammation, and cell survival^11^. It has been shown that PUFA composition changes in red blood cells (RBCs) reflect the composition changes in the brain in both primate and human studies^12,13^. Considering the accessibility and correlations with the brain, it is reasonable to obtain information about the brain from the measurement of fatty acids in RBCs to some extent. Reduced levels of PUFAs were found in postmortem brains^14^ and RBCs^15^ of patients with schizophrenia, though nonsignificant differences were also reported in PUFA levels in the corpus callosum of a small cohort of schizophrenia patients as compared with controls^16^. Our previous study showed that reduced fatty acid levels and disrupted biosynthesis pathways could be associated with the pat of schizophrenia^17^. It is hypothesized that the depletion of PUFAs is ascribed to an increased breakdown caused by elevated phospholipase A2 and lipid peroxidation rather than decreased incorporation into the membrane^18,19^.

Several lines of evidence have shown that atypical antipsychotic drugs (AAPDs) increase erythrocyte and brain PUFAs by augmenting biosynthesis^20^ or decreasing arachidonic acid turnover^21^ in animal studies. Increased delta-6 desaturase activity, decreased delta-5 desaturase activity, and increased stearoyl-CoA desaturase (SCD) activity were reported after treatment with AAPDs while assessed by product/precursor ratios^22–24^. However, the alteration in the elongation process of fatty acids seemed to be inconsistent in different pathways of the n-6 and n-3 families^25^.

The effects of atypical antipsychotic medication on membrane PUFAs have not been clarified in a large sample of patients with schizophrenia. The heterogeneity of published results causes great difficulty to define the PUFA composition changes in erythrocyte membranes due to the influences of sex, age, ethnicity, disease process, duration of illness, treatment, dietary habits, and smoking^26^. Among these factors, relapse has been proven to contribute to antipsychotic treatment resistance^3^. Thus, fatty acids were measured in RBCs of patients from multi-center with schizophrenia ranging from first-episode drug-naïve patients to chronic ones in the present study. The duration of illness was analyzed as a confounder. We aimed to explore the erythrocyte membrane PUFA alterations of patients under different episodes after atypical antipsychotic treatment as well as the relationship between FA changes and improvement in psychotic symptoms.

## Results

### Demographics

A cohort of 327 patients with schizophrenia and 159 healthy controls from 2016 to 2018 was recruited and the characteristics of the subjects are presented in Supplementary Table 1. There were no significant differences in sex or age between these two groups of participants. The characteristics of patients from each site are shown in Supplementary Table 2. Patients were divided into four subgroups according to the times of relapse at enrollment, and their characteristics are also shown in Supplementary Table 1. Nearly half of the patients were treated with risperidone or olanzapine, while 30% of the patients received combination treatment with AAPDs. The chlorpromazine-equivalent dose was calculated using the defined daily dose method^27^. Patients with 2–3 episodes received the largest doses, followed by the first-episode subgroup, although the difference was not statistically significant (*F*_3,__304_ = 2.204, *p* = 0.088).

### Symptom improvements among different episode patient subgroups

**Fig. 1:**
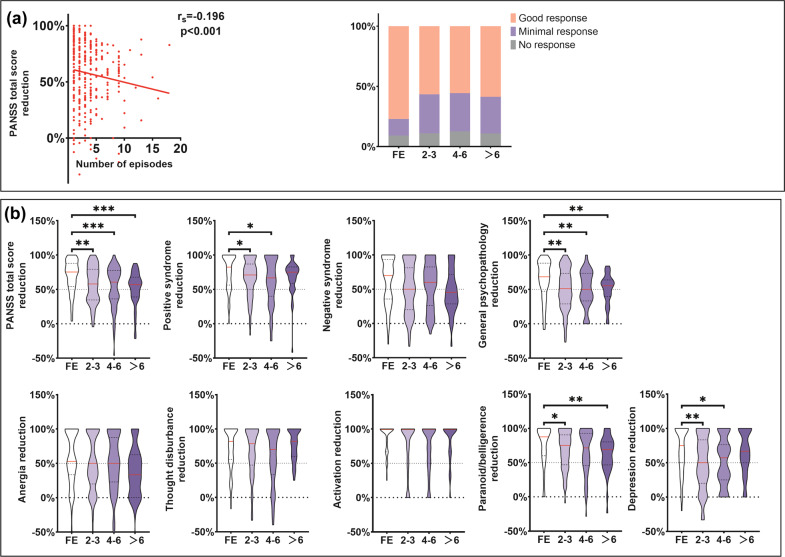
Symptom improvement after treatment among patients with different episodes. **a** correlation between PANSS total score reduction and number of episodes; proportion of treatment response in patient subgroups of different episodes. **b** PANSS reduction after treatment among patients with different episodes. First-episode patients showed the largest improvement in PANSS total scores, positive, general psychopathology, paranoid/belligerence, and depression. No significant differences were found in negative symptoms or other cluster scores (anergia, thought disturbance, and activation). FE, first episode; Spearman correlation, Kruskal–Wallis test and a Dunn–Bonferroni post hoc method were used; *p < 0.05, **p < 0.01, ***p < 0.001.

Significant decrease in PANSS total and subscale scores were observed in all patient groups with different episodes after treatment. The number of episodes is negatively correlated with the PANSS total score reduction (*r*_*s*_ = −0.196, *p* < 0.001) and the proportion of good response is the highest in FE group (Fig. 1a). First-episode patients showed the largest improvement in positive, general psychopathology, paranoid/belligerence, depression, and PANSS total score (Fig. 1b). We found no significant differences in negative symptoms, anergia, thought disturbance, or activation.

### Changes in membrane fatty acid levels and ratios in all patients with schizophrenia after antipsychotic treatment

**Fig. 2:**
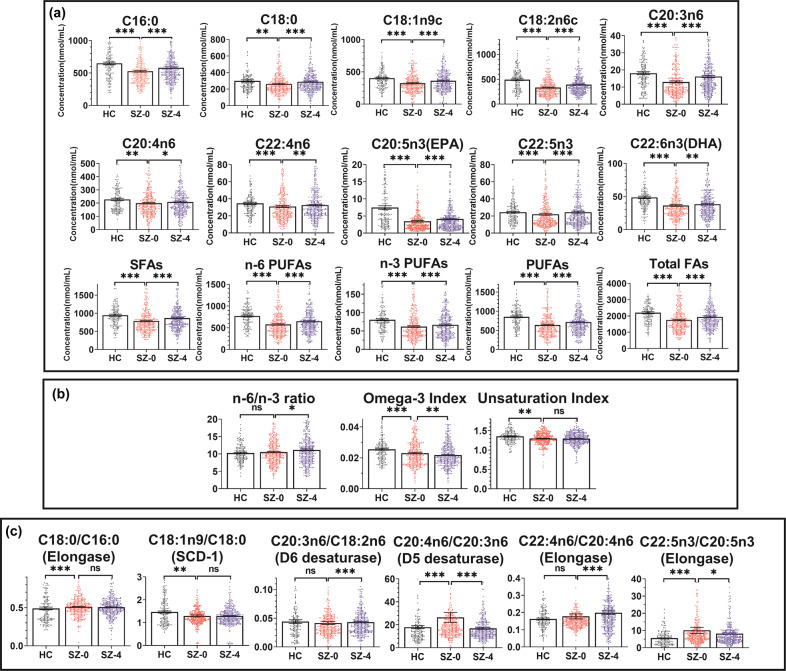
Differences in membrane fatty acid levels and ratios between patients with schizophrenia at baseline (SZ-0) and healthy controls (HC) as well as changes after antipsychotic treatment (SZ-4). **a** Specific fatty acids we measured and summations for SFAs, n-6 PUFAs, n-3 PUFAs, PUFAs, and total FAs; **b** n-6/n-3 ratio, omega-3 index and unsaturation index; **c** fatty acid ratios (markers of enzymes). SFAs, saturated fatty acids; PUFAs, polyunsaturated fatty acids; FAs, fatty acids; plots are shown as the means and standard errors; the Mann–Whitney test was used to analyze the variations between HC and SZ-0, and group pairwise differences between SZ-0 and SZ-4 were determined by the Wilcoxon signed rank test. *p < 0.05, **p < 0.01, ***p < 0.001.

Figure 2 shows that the levels of both saturated and unsaturated fatty acids were decreased in schizophrenia patients at baseline compared to healthy controls and increased after four weeks of antipsychotic treatment. No significant difference was observed for the ratio of n-6/n-3 PUFAs between the patients and the controls (Cohen’s *d* = 0, *Z* = −0.002, *p* = 0.999), while the ratio was increased after treatment (Cohen’s *d* = 0.218, *Z* = −2.388, *p* = 0.017). The omega-3 index, which indicates the ratio of EPA and DHA in total membrane fatty acid levels, was lower in the patients than in the controls (Cohen’s *d* = 0.311, *Z* = −3.389, *p* < 0.001) and decreased after treatment (Cohen’s *d* = 0.249, *Z* = −2.722, *p* = 0.006). UI was also lower in schizophrenia patients (Cohen’s *d* = 0.279, *Z* = −3.051, *p* = 0.002) but remained unaltered after treatment (Cohen’s *d* = 0.012, *Z* = −0.154, *p* = 0.878). A higher ratio of C18:0/C16:0 and a lower ratio of C18:1n9/C18:0 (SCD-1) were observed in the patient group compared with healthy controls (Cohen’s *d* = 0.314, *Z* = −3.421, *p* < 0.001; Cohen’s *d* = 0.269, *Z* = −2.935, *p* = 0.003) and these ratios showed no significant changes after treatment (Cohen’s *d* = 0.061, *Z* = −0.779, *p* = 0.436; Cohen’s *d* = 0.079, *Z* = −1.004, *p* = 0.315). For the ratios of C20:3n6/C18:2n6 (D6 desaturase) and C22:4n6/C20:4n6 (elongase), there were no significant differences between the SZ baseline and the controls (Cohen’s *d* = 0.035, *Z* = −0.382, *p* = 0.702; Cohen’s *d* = 0.139, *Z* = −1.531, *p* = 0.126), but these two ratios were upregulated after treatment (Cohen’s *d* = 0.261, *Z* = −3.312, *p* < 0.001; Cohen’s *d* = 0.363, *Z* = −4.572, *p* < 0.001). In addition, higher ratios of C20:4n6/C20:3n6 (D5 desaturase) and C22:5n3/C20:5n3 (elongase) in patients were downregulated by antipsychotic treatment (Cohen’s *d* = 0.523, *Z* = −6.471, *p* < 0.001; Cohen’s *d* = 0.162, *Z* = −2.068, *p* = 0.039).

### Changes in membrane fatty acid levels and ratios of different episode patient subgroups after antipsychotic treatment

**Fig. 3:**
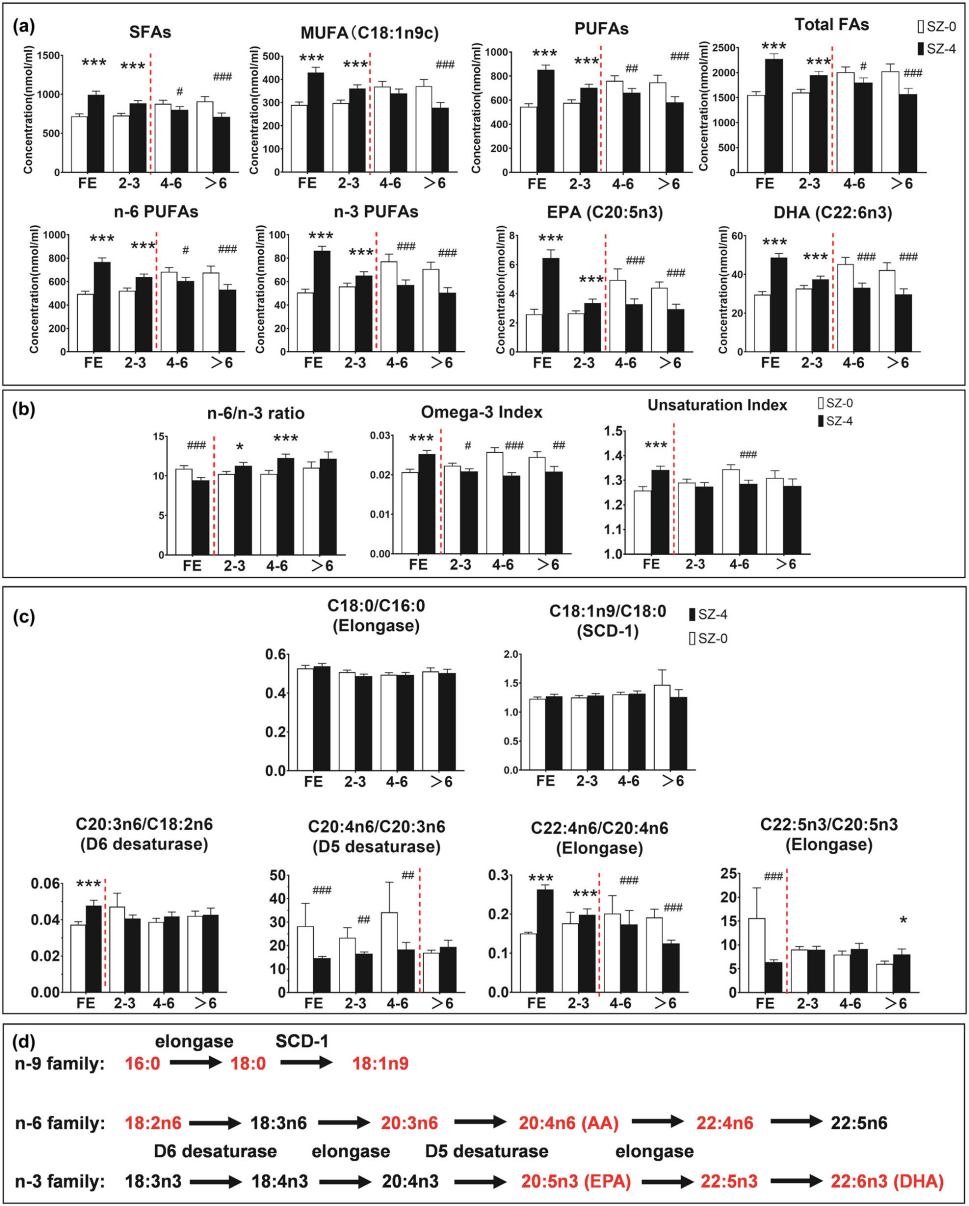
Differences in membrane fatty acid levels and ratios between baseline and after antipsychotic treatment in patient subgroups divided by episodes. **a** EPA (C20:5n3), DHA (C22:6n3) and summations for SFAs, MUFAs, PUFAs, n-6 PUFAs, n-3 PUFAs, and total FAs; **b** n-6/n-3 ratio, omega-3 index and unsaturation index. **c** Fatty acid ratios that present corresponding enzymes in the fatty acid synthetic pathways; **d** the synthetic pathways of fatty acids. SCD-1, stearoyl-coenzyme A desaturase-1. The fatty acids in red text indicate the types of specific fatty acids we analyzed in this study. The red dotted line divides the opposing trends of changes among the four subgroups. FE, first-episode; MUFAs, monounsaturated fatty acids; EPA, eicosapentaenoic acid; DHA, docosahexaenoic acid. Plots are shown as the means and standard errors. The Wilcoxon signed rank test was used. *p < 0.05, **p < 0.01, ***p < 0.001 for increase; #*p* < 0.05, ##*p* < 0.01, ###*p* < 0.001 for decrease.

According to the episodes at enrollment, the recruited patients were divided into four subgroups: first episode (FE), 2–3 episodes, 4–6 episodes, and over 6 episodes. We measured the concentrations of ten fatty acids in the erythrocyte membrane at baseline (SZ-0) and after four weeks of treatment (SZ-4), and the values are listed in Supplementary Table 3. Figure 3 describes the changes in membrane fatty acid levels and ratios after treatment, which show different trends among the subgroups. All fatty acid levels were increased after treatment in the groups of FE and 2–3 episodes (Fig. 3a and Supplementary Table 3). Interestingly, the levels of fatty acids were significantly decreased after treatment in the groups of 4–6 and over 6 episodes expect the levels of C18:1n9c, C18:2n6, and C20:3n6 PUFAs in the subgroup 4–6 episodes, which showed nonsignificant changes (Fig. 3a and Supplementary Table 3). The n-6/n-3 ratio declined only in the FE group (Cohen’s *d* = 0.546, *Z* = −3.512, *p* < 0.001) and rose in patients with 2–3 and 4–6 episodes (Fig. 3b). In the subgroup of over 6 episodes, n-6/n-3 ratio showed no significant changes but a trend of increasing. Consistently, the omega-3 index and UI were increased in the FE group and decreased in relapsed patients. As for the ratios of fatty acid product-to-precursor, C18:0/C16:0 and C18:1n9/C18:0 (SCD-1) had no significant changes after treatment among all the subgroups (Fig. 3c). An increased ratio of C20:3n6/C18:2n6 (D6 desaturase) was observed in first-episode patients (Cohen’s *d* = 0.662, *Z* = −4.192, *p* < 0.001) but not in relapsed patients. The C20:4n6/C20:3n6 ratio (D5 desaturase) was decreased after treatment in subgroups except the group of over 6 episodes (Cohen’s *d* = 0.067, *Z* = −0.328, *p* = 0.743). The ratio of C22:4n6/C20:4n6 (elongase) was upregulated in patients within 3 episodes after treatment but downregulated in patients with over 3 episodes. We observed a decreased ratio of C22:5n3/C20:5n3 (elongase) only in the FE group (Cohen’s *d* = 0.941, *Z* = −5.681, *p* < 0.001), and the ratio was significantly increased in the patient group of over 6 episodes (Cohen’s *d* = 0.473, *Z* = −2.256, *p* = 0.024).

### Duration of illness as a confounder of membrane fatty acid metabolism in more-responsive and less-responsive subgroups after antipsychotic treatment

According to the different trends of changes in membrane fatty acid levels after antipsychotic treatment between patients with 1–3 episodes and those with over 3, we divided the patients into two subgroups: more-responsive (1–3 episodes) and less-responsive (over 3 episodes) subgroups. In each subgroup, patients were divided by duration of illness (0–5 years, 5–10 years, and over 10 years), and the characteristics are shown in Supplementary Table 4. Trends of changes in fatty acid levels after antipsychotic treatment remained consistent, as the patients underwent longer disease courses in both more-responsive and less-responsive subgroups (Supplementary Fig. 1). Generally speaking, membrane fatty acids were increased in patients within 3 episodes, while they were decreased in patients with over 3 episodes. It suggested that relapse outweighed disease progression as a confounding factor of the effects of antipsychotic drugs on membrane fatty acid metabolism. The ratio of n-6/n-3 PUFAs was increased in the less-responsive subgroup but it showed nonsignificant changes in more-responsive patients. Increased omega-3 index and UI were only found in patients with a disease duration of 0–5 years in the more-responsive group. Consistent with previous analyses, the ratio of C18:0/C16:0 showed nonsignificant changes. C18:1n9/C18:0 was slightly increased only in patients with a disease duration of 5–10 years in the more-responsive subgroup (Cohen’s *d* = 0.559, *Z* = −2.316, *p* = 0.021). Increased C20:3n6/C18:2n6 (D6 desaturase) was found only in the patients with a disease duration of 0–5 years in the more-responsive subgroup (Cohen’s *d* = 0.463, *Z* = −3.784, *p* < 0.001), indicating the possible effects of disease course. Decreased C20:4n6/C20:3n6 ratio (D5 desaturase) was observed in both more-responsive and less-responsive subgroup. The ratio of C22:4n6/C20:4n6 (elongase) was increased in the more-responsive subgroup and decreased in the less-responsive subgroup. A reduced ratio of C22:5n3/C20:5n3 (elongase) was observed only in patients with a disease duration of 0–5 years in the more-responsive subgroup (Cohen’s *d* = 0.589, *Z* = −4.745, *p* < 0.001).

### Correlations between fatty acid level changes and symptom improvement

**Fig. 4:**
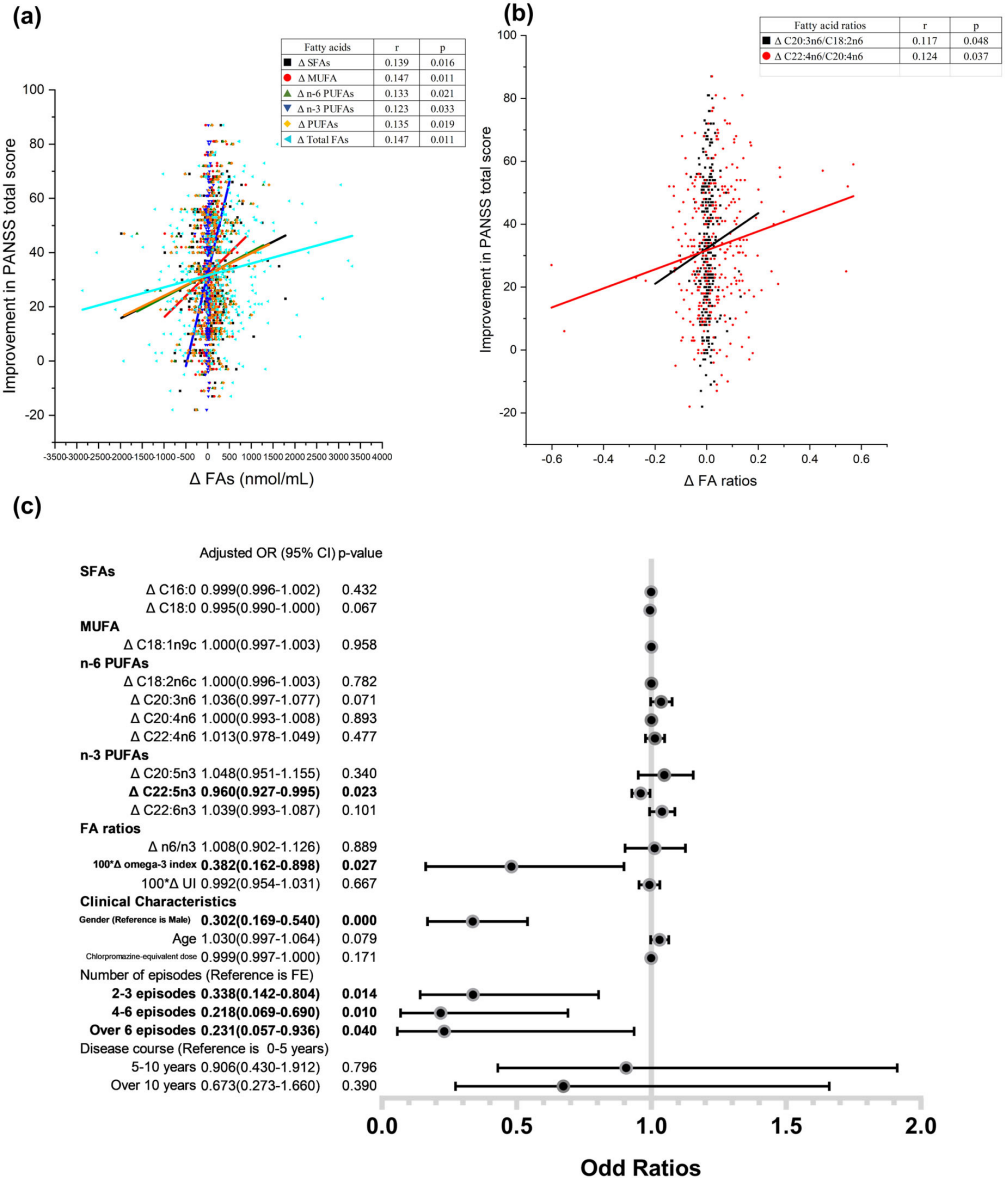
Partial correlation and logistic regression analyses in all the patients with schizophrenia after antipsychotic treatment. **a** Significant correlations between fatty acid level changes and PANSS total score improvement. **b** Significant correlations between fatty acid ratio changes and PANSS total score improvement. **c** Forest plot of logistic regression of treatment response and fatty acid level changes. Gender, age, chlorpromazine-equivalent dose, number of episodes, and disease course are added to the regression model to control the influence of these confounding factors on antipsychotic responses. Partial Spearman’s rank correlation and binary logistic regression were used.

Two-tailed partial correlation analysis controlling for gender, age, and chlorpromazine-equivalent dose were performed between the change values of fatty acid levels and ratios and the improvement in symptomatology among total schizophrenia patients as well as four subgroups of different episodes. Improvement in PANSS total score was positively correlated with Δ SFAs (*r* = 0.139 *p* = 0.016), Δ MUFA (*r* = 0.147, *p* = 0.011), Δ n-6 PUFAs (*r* = 0.133, *p* = 0.021), Δ n-3 PUFAs (*r*_*s*_ = 0.123, *p* = 0.033), Δ PUFAs (*r* = 0.135, *p* = 0.019), Δ total FAs (*r* = 0.147, *p* = 0.011) (Fig. 4a). Besides, improvement in PANSS total score was positively correlated with Δ C20:3n6/C18:2n6 (D6D) and Δ C22:4n6/C20:4n6 (Fig. 4b). The partial correlations between PANSS subscale improvement and change values of fatty acids and ratios for total patients as well as subgroups are also performed controlling for sex, age and chlorpromazine-equivalent dose as shown in Supplementary Fig. 2.

The logistic regression model (Fig. 4c) showed the correlation between fatty acid level changes and treatment response in total patients with schizophrenia after controlling for sex, age, chlorpromazine-equivalent dose, number of episodes, and disease duration. The Hosmer–Lemeshow test indicated that the model was a good fit (*p* = 0.870). The level change of C22:5n3 and ΔOmega-3 index were negatively correlated with treatment response (*Adjusted OR* 0.960, *95% CI* 0.927–0.995, *p* = 0.023; *Adjusted OR* 0.382, *95% CI* 0.162–0.898, *p* = 0.027). Female gender was a significant risk factor for poor treatment response (*Adjusted OR* 0.302, *95% CI* 0.169–0.540, *p* < 0.001). Number of episodes were negatively correlated with treatment response, but no significant correlation was observed in disease duration.

## Discussion

In this study, we reported the different trends of changes in erythrocyte membrane fatty acid levels in schizophrenia patients with different episodes after antipsychotic treatment. The levels of all erythrocyte membrane fatty acids were increased after atypical antipsychotic treatment in schizophrenia patients as a total group. However, antipsychotic drugs seemed to gradually lose their efficacy of upregulating fatty acid levels, especially n-3 PUFAs, in patients with multiple episodes. Subgroup analyses suggest that the third episode was deemed a turning point toward the opposite effects (Fig. 3). The dysregulation of n-3 PUFAs, especially EPA and DHA, preceded that of n-6 PUFAs. Failure to increase D6 desaturase activity and decrease D5 desaturase activity induced by antipsychotic treatment is responsible for the opposite effects in patients with multiple episodes. Additionally, fatty acid level changes after treatment showed a positive correlation with symptom improvements in total schizophrenia patients, especially general psychopathology and depression. Increased C20:3n6/C18:2n6 and C22:4n6/C20:4n6 were correlated with symptom improvements. Thus, the results suggest that dysregulation of erythrocyte membrane fatty acid levels is a strong indicator of diminished treatment response in schizophrenia after several relapses.

Consistent with previous reports^15^, we observed lower levels of PUFAs in the erythrocyte membrane of schizophrenia patients at drug-naïve baseline than in healthy controls. Saturated, monounsaturated, and total fatty acid concentrations were also decreased in schizophrenia, even though no significant differences were reported in other studies with smaller cohorts of patients^28^. Discrepancies in saturated fatty acid levels could be due to different sample sizes and the heterogeneity of patients (eg. first episode or multi-episode patients). Nonetheless, the abnormalities of fatty acids were reversed significantly by four weeks of atypical antipsychotic treatment. In a clinical study of patients with chronic schizophrenia, concentrations of membrane fatty acids are not changed after antipsychotic treatment except for DHA with a slight increase^29^. We assume that relapse could account for the inconsistency. Subgroup analysis was performed to explore the effect of relapse on erythrocyte membrane fatty acid levels, indicating different patterns of treatment response between first-episode and multi-episode patients. Increased levels of saturated, monounsaturated, and PUFAs decreased gradually with the increasing relapse, and the cut-off event occurred after the third episode (Fig. 3a). It seemed that antipsychotic drugs lost their effects on the normalization of fatty acid metabolism with the relapse of the disease. Moreover, n-3 PUFAs seemed to be maladjusted earlier than n-6 PUFAs since the reduction in n-3 PUFAs appeared in the patient group of 4–6 episodes, while the reduction of C18:2n6c and C20:3n6 appeared in the subgroup of over 6 episodes. Consequently, the ratio of n-6/n-3 PUFAs was significantly lowered in first-episode patients but elevated in multi-episode patients after antipsychotic treatment. Saturated fatty acids and n-6 PUFAs are viewed as pro-inflammatory molecules, whereas n-3 fatty acids are viewed as anti-inflammatory factors^30^. Therefore, a high ratio of n-6/n-3 PUFAs maintains the physiological state in a pro-inflammatory state, which induces psychiatric symptoms by interfering with the functions of various neurotransmitters, such as the dopaminergic system and glutamatergic neurotransmission^31,32^. A low omega-3 index in patients was observed in our study, but was increased after treatment in first-episode patients. EPA and DHA are fundamental to brain function and show neuroprotective properties^33^. The downregulation of the omega-3 index may be responsible for treatment resistance in patients with multiple episodes. The UI was reported to be negatively correlated with the stability of the erythrocyte membrane^34^. Changes in membrane fluidity can affect physiological functions via regulation of the densities and binding affinities of neurotransmitters, as well as the properties of membrane proteins^35,36^. Decreased membrane fluidity has been reported in drug-free patients with schizophrenia, but haloperidol treatment had no effect on membrane fluidity^36^. The perturbation of membrane fluidity may result in altered neurotransmission in schizophrenia. In our study, an increased UI in first-episode patients after treatment presented higher membrane fluidity induced by AAPDs, which may contribute to the treatment of schizophrenia. Thus, a lower UI in multi-episode patients after treatment indicates lower membrane fluidity, which can be involved in treatment resistance due to disturbed neurotransmission. This is consistent with the findings that drug-free patients with decreased membrane fluidity may be prone to relapse ^36^.

Alterations in fatty acids could result from disturbances in the biosynthesis pathway. The product/precursor ratios can be used to estimate the enzyme activity in the process. In the current study, higher C18:0/C16:0 ratios and lower C18:1n9/C18:0 ratios were observed in schizophrenia patients compared to healthy control subjects, while neither of them changed significantly after treatment in all patients or different episode subgroups. The C18:1n9/C18:0 ratio provides an estimated index of SCD1 that catalyzes the conversion of the saturated fatty acid C18:0 to the monounsaturated fatty acid C18:1n9. This finding of unchanged SCD1 activity is in agreement with one preclinical study^24^ but contrasts with the results of quantitative real-time PCR, which found that olanzapine significantly upregulated SCD expression in whole blood compared to unmedicated patients ^37^.

The key enzymes in the PUFA synthetic pathways are D6 desaturase, D5 desaturase, and elongases. N-6 and n-3 PUFAs are known to compete for desaturation enzymes, and both D6 desaturase and D5 desaturase prefer ALA (C18:3n3, alpha-linolenic acid) to LA (C18:2n6, linoleic acid). It was reported that antipsychotic medications increased liver D6 desaturase mRNA expression in rats^23^. The reduced plasma index of D5 desaturase (C20:4n6/C20:3n6) was investigated in mice treated with olanzapine for 8 weeks^24^. In our study, the variation in the C20:3n6/18:2n6 ratio indicated that antipsychotic treatment increased D6 desaturase activity in first-episode rather than in multi-episode patients (Fig. 3c). The change in the C20:4n6/C20:3n6 ratio after treatment indicated that the cut-off of decreased D5 desaturase activity did not appear until 7 episodes. Our results suggest that the effects of AAPDs on PUFA biosynthesis could be undermined in the face of relapse. D6 desaturase was more vulnerable than D5 desaturase to relapse of the disease. The ratios of C22:4n6/C20:4n6 and C22:5n3/C20:5n3 were used to estimate the elongation process of n-6 and n-3 PUFA family, respectively. The increasing effect on the C22:4n6/C20:4n6 ratio was reversed after 4 episodes, while the decreased C22:5n3/C20:5n3 ratio in first-episode patients became nonsignificant after 2 episodes. It is reasonable to hypothesize that antipsychotic drugs may fail to regulate fatty acid metabolism with increased numbers of episodes, especially the n-3 PUFA family. We wondered if this contributed to treatment resistance in chronic patients receiving antipsychotic drugs.

Evidence shows that relapse is associated with disease progression in schizophrenia with a longer response time, persistent residual negative symptom, refractory treatment, and pathological changes in brain structure^38^. Since more relapses are accompanied by a longer duration of disease, we analyzed the effects of antipsychotic treatment on membrane fatty acids in patient groups with different durations of disease. Our results indicate that the dysregulation of membrane phospholipids occurs in patients with over 3 episodes rather than those with longer durations of illness within 3 episodes (Supplementary Fig. 1). The result of logistic regression also showed that the number of episodes was an independent factor of treatment response but the result of disease duration was not significant (Fig. 4c). Prevention of relapse should be given more attention to achieve drug efficacy.

We were unable to reproduce the associations between PUFA levels and negative symptoms^39^, but we did find some interesting associations between fatty acid deficits and the improvement in psychotic states. More specifically, the changes in both SFA and PUFA levels were positively correlated with the improvement in the general psychopathology subscale and depression (Supplementary Fig. 2). PUFA metabolism has been reported as a potential biomarker for major depressive disorder^40^. For depressive patients with high inflammation, EPA supplementation had medium treatment effect size for improvement in depression ratings^41^. Similarly, the depression subscale of schizophrenia may be improved by n-3 PUFA supplementation via an anti-inflammatory mechanism. The logistic regression model showed that gender, number of episodes, the changes of C22:5n3 (DPA) concentration, and omega-3 index were significant risk factors correlated with treatment responsiveness. As the direct metabolite of EPA, decreased DPA levels in RBCs were found to be associated with schizophrenia syndromes^26^. These results indicate that the remission of psychiatric symptoms is involved in the upregulation of membrane fatty acids, especially n-3 PUFAs. Moreover, it has been reported that disease severity in treatment-resistant schizophrenia (TRS) is mainly affected by negative symptoms^42^. Reduced serum omega-3 fatty acids were associated with cognitive impairment in patients with schizophrenia^43^. From clinical perspective, the present findings suggest the possible therapeutic effect of omega-3 PUFA supplementation for patients with multi-episode schizophrenia, especially those with severe negative symptoms and depression.

Sex differences in schizophrenia are one of the most consistently reported aspects of the illness and are described in almost all features like prevalence, incidence, age at onset, clinical presentation, course, and the response to treatment^44^. Overall, women tend to respond better to antipsychotic treatments, and require lower doses than men, however, this is not definite and dependent upon the type of antipsychotics, menopausal status, and disease progression^44^. In the present cohort of schizophrenia patients, women and men are not different in BMI, number of episodes, duration of disease, and drug intake, except age (Supplementary Table 5). However, women seem to fair worse in response than men as shown by our data, and this may partly due to the menstrual phase when we recruited. Since the steroid hormones can change drastically throughout the menstrual cycle and psychotic symptomatology varies with menstrual phases^45^, for female subjects, we inquired about the date of their last menstrual bleeding and restricted the sampling time to the period within the early follicular phase (Day1–7), which makes them have relatively lower estrogen levels. Accumulated evidence suggests that higher symptom levels, higher rates of hospital admission, and poorer cognitive performance are associated with lower estrogen levels^45,46^. However, the functional mechanisms underlying the sex difference in treatment response are very complex and remain unclear in human studies, which requires further investigations with larger sample size and more specific design.

Depletion of PUFAs has previously been reported to be associated with lipid peroxidation and inflammatory processes in the pathobiology of schizophrenia^47^. Since erythrocyte membrane fatty acid composition is regulated by liver biosynthesis, phospholipase a2 (PLA2) activity, and lipid peroxidation, multiple pathways are involved in the dysregulation of membrane phospholipids. Antipsychotic drugs increased rat erythrocyte DHA (docosahexaenoic acid, 22:6n-3) concentration, but not all antipsychotic medications augmented PUFA biosynthesis^23^. Among the five antipsychotic drugs they studied, only risperidone and paliperidone increased liver D6 desaturase (Fads2) mRNA expression. It has been found that increased phospholipase A2 activity and enhanced lipid peroxidation cause the reduction in PUFAs, which decreases the anti-inflammatory and neuroprotective properties^15^. Arachidonic acid (AA) released from membrane phospholipids via the activation of PLA2 has been implicated in the pathogenesis of schizophrenia^19^. Increased serum calcium-independent phospholipase A2 (iPLA2) activity has been found in unmedicated first-episode schizophrenia patients but not in multi-episode chronic patients^48^. An 8-week study showed that antipsychotic medications reduced the high iPLA2 activity of patients with schizophrenia^49^. From another angle, enhanced lipid peroxidation caused by changes in enzymatic and non‐enzymatic antioxidant systems is also responsible for the lower PUFA levels in schizophrenia^50^. In support, several studies have reported that AAPDs restore PUFAs by the ability of inhibiting lipid peroxidation^51^. Higher plasma thiobarbituric acid reactive substance levels were reported in never-medicated patients with schizophrenia^52^. There was a significant increase in serum malondialdehyde (MDA) in schizophrenic patients compared to control subjects and AAPDs significantly altered the trend ^53^.

There are two distinct types of treatment resistance: early treatment resistance (from onset) and late treatment resistance (at a later stage)^54^. TRS implicates dysregulation in neurotransmitters, particularly dopamine and glutamate^55^. Several lines of evidence suggest that the dopaminergic system may be involved in treatment resistance, such as dopamine supersensitivity^56^ and hypofunctional dopamine uptake^57^. Compared to non-TRS, patients with TRS showed reduced striatal dopamine synthesis and increased glutamate levels in the anterior cingulate cortex based on neuroimaging results^58^. Glutamatergic dysfunction seemed to play an important role in treatment resistance to conventional antipsychotic drugs. Higher levels of PLA2 have been reported to be positively correlated with the disease duration and number of episodes^59^. Elevated anti-gliadin IgG antibodies are related to treatment resistance in schizophrenia^60^. Thus, an inflammatory state may contribute to the poor drug response in the patients with multi-episode schizophrenia. In addition, increased lipid peroxidation^61^ was reported in patients with TRS. In this study, our-findings expand the knowledge that disordered fatty acid metabolism may be responsible for the poor drug response in multi-episode patients compared to first-episode patients, especially for the dysregulation of desaturases.

This is a multicenter real-world study with data collected in routine clinical practice. There are several limitations to be mentioned in this study. First, some confounding factors, such as dietary habits, medication compliance, smoking, and metabolic state, might affect the reliability of the results. To minimize the influence of these factors, we recruited patients in the hospital with great medication compliance as well as standard meals, and we excluded patients with metabolic disorders such as diabetes. To control the influence of diet before the research, the patients and healthy controls were recruited from the same area. Therefore, their standard of living as well as dietary habits were generally comparable. Second, it has not been identified whether treatment resistance is the cause or the outcome of relapse in this research. A vicious cycle may exist in this situation. Several follow-up studies have reported that antipsychotic treatment response is reduced in subsequent episodes following effective treatment in first-episode patients^3,62^, which supports our hypothesis that relapse may contribute to treatment resistance in schizophrenia. Third, we performed multiple comparisons without adjustment to avoid missing important findings in this exploratory analysis. Fourth, although product-to-precursor ratios of fatty acids may not necessarily reflect differences in the pathways, it is by far the most representative way to use the peripheral fatty acid levels to indirectly estimate desaturase activity in schizophrenia patients^63^. There are many other studies making the same estimations, which are largely in support of our theory and results^28^. Besides, elevated delta-6 desaturase (FADS2) expression in the postmortem prefrontal cortex of schizophrenia patients has also been reported, which suggested that the changes of fatty acid desaturase could be paralleled in the brain and in RBCs of schizophrenia^64^. Since the access of living human brain tissue is not ethical, to confirm the effects of antipsychotic drugs on fatty acid desaturase, further studies are warranted to focus on animal study. Tools like RT-qPCR can be used to analyze the expression of desaturase in animal brain and peripheral RBCs.

In summary, we found that erythrocyte membrane fatty acid levels and ratios were altered differently in patients with different episodes after treatment. Fatty acid desaturase and elongase dysregulation may play a significant role in the reduced treatment response of multi-episode patients. Further researches into the detailed mechanisms of treatment resistance, involving fatty acid metabolism, neurotransmission, inflammation, and oxidative stress, are warranted.

## Methods

### Participants

**Fig. 5:**
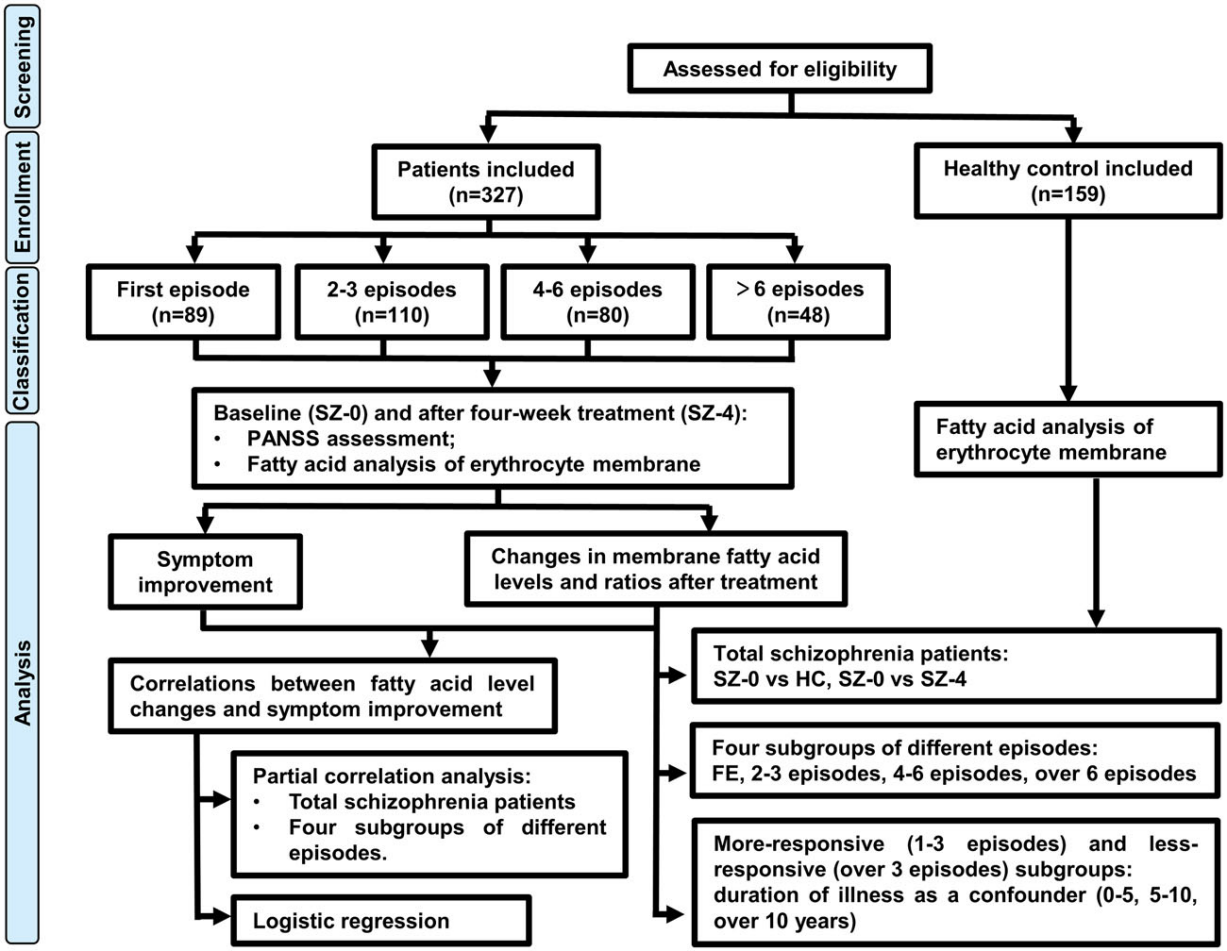
Flowchart of the research. Through eligibility assessment, 327 schizophrenia patients and 159 healthy controls were recruited. The recruited patients were divided into four subgroups according to episodes: first episode (FE), 2–3 episodes, 4–6 episodes, and over 6 episodes. We measured the concentrations of ten fatty acids in the erythrocyte membrane and performed PANSS assessment at baseline (SZ-0) and after four weeks of treatment (SZ-4). Total and subgroup analyses were conducted to identify differential biomarkers between schizophrenia patients and healthy controls. Correlations between fatty acid level changes and symptom improvement were analyzed by partial correlation analysis and logistic regression, to explore potential biomarkers indicative of treatment responsiveness.

The experimental protocol was registered in the Chinese Clinical Trials Registry (http://www.chictr.org.cn/) as ChiCTR-OOC-16008988 and approved by the Ethics Committee of the Second Xiangya Hospital of Central South University, the Second People’s Hospital of Hunan Province, and Changsha Psychiatric Hospital. Written informed consent was obtained from the participants or their legal guardians. Patients aged 15–60 years were recruited at the above mentioned three centers (see Fig. 5 for flowchart). All participants were Han Chinese who met the following inclusion criteria: (1) DSM-5 criteria for schizophrenia, schizoaffective disorder, or schizophreniform disorder; and (2) newly hospitalized with first-onset, or recurrent psychosis and without taking antipsychotic drugs for more than one month prior to admission. We excluded patients complicated with Alzheimer’s disease, epilepsy, alcohol or drug abuse, or other serious physical or mental disorders. Patients who were taking medicine for hyperlipidemia or diabetes mellitus were also excluded. For female subjects, we restricted the sampling time to the period within the early follicular phase (Day1–7) to control the influence of steroid hormones. Antipsychotic medication strategy and dose were determined by experienced psychiatrists who conducted our initial screening process, complying with a standardized antipsychotic drug protocol until recovery. After recruitment, the patients were receiving atypical antipsychotic monotherapy or combination treatment and combination treatment includes all the ways in which one medication may be added to another^65^. All patients were served a standard diet from the hospital cafeteria and meal composition is listed in Supplementary Table 6.

We recruited healthy control subjects (HC group) who matched the patients in age and sex from the same area. Healthy controls had no history of mental disorders and no first- or second-degree relatives with psychotic illnesses.

### Clinical assessments

At enrollment and after 4 weeks of treatment, patients were rated on the positive and negative syndrome scale (PANSS) performed by three senior experienced psychiatrists^66^. They were trained in administering the PANSS, and good inter-rater reliability was established. The PANSS total score, three scales (positive syndrome, negative syndrome, and general psychopathology) and five cluster scores (anergia, thought disturbance, activation, paranoid belligerence, depression) were used to assess the psychopathological states^67^. PANSS reduction was calculated to compare the symptom improvement among these subgroups using the formula [(SZ-0–SZ-4)/SZ-0]×100%. “Minimal response” was defined as at least a 20% PANSS reduction from baseline and “good response” at least a 50% PANSS reduction^68^. Relapse was defined by any one of the following: psychiatric hospitalization; an increase in the level of psychiatric care and 25% PANSS total score increase; deliberate self-injury; suicidal or homicidal ideation that was clinically significant in the investigator’s judgment; violent behavior resulting in clinically significant damage to another person or property^69^.

### Sample collection and preparation

Fasting blood samples were collected in the morning (7 am) at baseline and after 4 weeks of antipsychotic treatment for patients. Healthy controls were examined only once. The fasting blood samples were drawn into 5 mL vacutainer tubes containing EDTA, and then the blood was centrifuged for 5 min (3000 r/min). The RBCs in the lower layer were transferred into Eppendorf tubes and stored at −80 °C until GC/MS analysis.

### Analysis of fatty acids in erythrocyte membranes

A method for lipid extraction and purification, as well as GC-MS analysis, were carried out following a method modified from a previously described procedure^70^. Briefly, 450 μL of packed erythrocytes was placed in a 5 mL clean glass centrifuge tube. After mixed with 450 μL of chilled water first, the erythrocytes were then slowly added with 1.5 mL of isopropanol and 1 mL of chloroform during vortex-mixing, and washed by 1 mL 0.1 M KCl solution for 3 min. After centrifugation at 3000 × *g* for 5 min, the top aqueous layer was abandoned and the bottom organic layer was further washed by 1 mL 0.1 M KCl solution for two more times. Then, 300 μL of the bottom organic layer was transferred to another 5 mL clean glass centrifuge tube, treated with 10 μL of 100 μM dibutyl hydroxy toluene solution in chloroform to prevent oxidation, and evaporate to dryness under a gentle stream of nitrogen. The dried powder was resuspended with 240 μL of hexane containing 0.15 μM internal standard (C17:0, heptadecanoic acid) and then mixed with 2 mL of 0.5 M KOH-methanol solution and 3 mL of 12.5% H_2_SO_4_-methanol solution under 60 °C water bath for 1.5 h, in order to covert the fatty acids to methyl esters. When the derivatization was completed, the mixture was vortexed with 2 mL of hexane and 1 mL of saturated NaCl solution for 3 min and then allowed to cool down and separate into three layers in 10 min. 1 mL of the top organic layer was aspirated into an injection vial.

After the preparation, 1 μL of the resulting fatty acid methyl esters (FAMEs) sample was run under a splitless injection mode on an Agilent 7890A/5975C GC-MS system. The capillary column for separation was VF-23 ms (Agilent): 30 m (length), I.D. 0.25 mm wide bore, and a film thickness of 0.25 μm. GC was programmed under the following temperature gradient: initial time at 50 °C for 1 min, 25 °C/min from 50 to 100 °C, 8 °C/min from 100 to 140 °C, 2 °C/min from 140 to 200 °C, finally 40 °C/min from 200 to 240 °C and holding at 240 °C for 4 min, with helium as the carrier gas. Injector and detector temperatures were both 250 °C. Peaks were identified by their times of authenticated FAME standards and characteristic mass spectra on the mass spectrometer. Analysis was performed in electron ionization mode (ionization energy, 70 eV; solvent delay, 2 min; MS quadruple temperature, 150 °C; source temperature, 250 °C). A calibration curve was obtained under the same experimental conditions with a standard FAME mixture (Supelco 37, Sigma-Aldrich, Shanghai, China) to correct differences in the detector response. The calibration curves were linear in the range of lipid phosphorus analyzed (*r* > 0.99) and with the coefficient of variance ranging from 2 to 6%. Quality controls (QCs) were inserted into each batch and distributed evenly in the sample sequences. The relative standard deviation of each analyte in QCs was as follows: 8.7% for C16:0, 7.7% for C18:0, 9.6% for C18:1n9c, 6.8% for C18:2n6c, 9.3% for C20:3n6, 8.2% for C20:4n6, 8.0% for C20:5n3, 10.7% for C22:4n6, 11.8% for C22:5n3 and 8.6% for C22:6n3, respectively.

The concentrations of fatty acids (mmol/mL packed RBC) were expressed as the mean ± standard error of the mean (SEM). The sum of n-6 fatty acids was C18:2n6c + C20:3n6 + C20:4n6 + C22:4n6. The sum of n-3 fatty acids was C20:5n3 + C22:5n3 + C22:6n3. The sum of n-3 and n-6 fatty acids was named PUFAs. The ratio of n-6/n-3 was calculated to evaluate the balance of the n-6 and n-3 families. The omega-3 index was calculated as the proportion of EPA + DHA levels in total fatty acids. To evaluate the average number of double bonds per FA molecule, the unsaturation index (UI) was calculated as Σ Ni × Mi, where Ni is the number of carbon–carbon double bonds of FA and Mi is the mole percentage^71^. Desaturase activity was estimated indirectly using the fatty acid product-to-precursor ratios, called the desaturase indexes^63^ (Fig. 3d). D5 desaturase activity was calculated as the ratio of C20:4n6/C20:3n6. And we used the ratio of C20:3n6/C18:2n6 to roughly estimate the D6 desaturase activity^28^. SCD1 activity was calculated as the ratio of C18:1/C18:0. The elongation process of n-6 and n-3 fatty acids was estimated using the C22:4n6/C20:4n6 ratio and C22:5n3/C20:5n3 ratio, respectively.

### Statistical analysis

The results were analyzed using SPSS version 18.0 software. Chi-square test was used to compare the gender differences of different groups. For all variables of fatty acid levels and ratios, a test for the hypothesis of normality was rejected for one or more of the groups at *p* < 0.05. Common transformations did not alter this conclusion. Hence, the data were found to be non-normally distributed. We used raw data of actual quantitative concentrations of the fatty acids to perform all the analysis without any data normalization or scaled data. The Mann–Whitney test was used to analyze the variations in independent samples, and group pairwise differences were determined by the Wilcoxon signed rank test. A *p*-value of less than 0.05 was considered statistically significant. Two-tailed partial correlation analysis was used to evaluate possible associations between fatty acid level changes and PANSS score improvement. A logistic regression model was fitted for treatment response, using fatty acid changes as the explanatory variables, and controlling for age, gender, drug intake, number of episodes, and disease duration. Hosmer–Lemeshow test was used to determine the goodness of fit. Treatment response was divided into poor response (PANSS reduction from baseline <50%) or good response (PANSS reduction from baseline ≥50%), and the odd ratios reflect the association between the extent of fatty acid changes and poor response/good response risk.

### Reporting summary

Further information on research design is available in the Nature Research Reporting Summary linked to this article.

## Supplementary information

Supplementary

Reporting Summary

## Data Availability

The data that support the findings of this study are available from the corresponding author upon reasonable request.

## Code availability

No non-standard code or algorithms were used in the generation of the results in this article.
