# Supplementary material for: Reduced erythrocyte membrane polyunsaturated fatty acid levels indicate diminished treatment response in patients with multi- versus first-episode schizophrenia
Source: Schizophrenia (Heidelb). 2022 Feb 25;8(1):7. doi: 10.1038/s41537-022-00214-2 (PMC8881498; doi:10.1038/s41537-022-00214-2)
Supplement: Supplementary file 1 — Supplementary [file 41537_2022_214_MOESM1_ESM.pdf]

Supplementary Table 1 Clinical characteristics of the recruited patients and healthy controls

Characteristics	Patients	HC	P-value	FE	2-3 episodes	4-6 episodes	Over 6 episodes	P-value
**N**	327	159		89	110	80	48	
**Male (%)**	147 (45.0%)	76 (47.8%)	0.555	44 (49.4%)	47 (42.7%)	31 (38.8%)	25 (52.1%)	0.366
**Age (years)**	32.70±10.40	32.86±10.09	0.879	28.97±11.22	31.13±8.95	35.17±8.53	39.13±11.04	< 0.001
**Range**	15-59	15-60	-	15-59	15-57	19-56	16-57	-
**BMI (kg/m^2)**	21.97±3.69	22.10±2.88	0.684	20.59±2.89	21.67±3.53	22.87±3.92	23.49±4.03	< 0.001
**Duration of disease (years)**	7.8±7.7	-	-	1.5±1.9	6.8±6.3	9.9±5.5	16.8±9.0	< 0.001
**Chlorpromazine-equivalent dose**	385.4±186.1	-	-	382.0±175.0	419.5±191.6	354.6±189.2	359.0±179.2	0.088
**AAPDs (n, %)**								
**Risperidone**	74 (22.63%)	-	-	23 (25.84%)	20 (18.18%)	17 (21.25%)	14 (29.17%)	-
**Olanzapine**	72 (22.02%)	-	-	26 (29.21%)	28 (25.45%)	12 (15.00%)	6 (12.50%)	-
**Aripiprazole**	28 (8.56%)	-	-	4 (4.49%)	14 (12.73%)	7 (8.75%)	3 (6.25%)	-
**Amisulpride**	16 (4.89%)	-	-	8 (8.99%)	4 (3.64%)	3 (3.75%)	1 (2.08%)	-
**Clozapine**	16 (4.89%)	-	-	1 (1.12%)	2 (1.82%)	9 (11.25%)	4 (8.33%)	-
**Others**	23 (7.03%)	-	-	6 (6.74%)	12 (10.91%)	4 (5.00%)	1 (2.08%)	-
**Combination treatment**	98 (29.97%)	-	-	21 (23.60%)	30 (27.27%)	28 (35.00%)	19 (39.58%)	-

Values represent as mean ± standard deviation; HC, healthy controls; FE, first episode; BMI, body mass index.

Supplementary Table 2 Demographic characteristics of patients from each clinical site

Characteristics	A	B	C	p
n	43	228	56	
Male (%)	23 (53.5%)	104 (45.6%)	20 (35.7%)	0.198
Age (years)	25.30±7.94	32.94±10.24	37.41±9.76	<0.001
BMI (kg·m-2)	20.08±2.72	21.91±3.70	23.30±3.70	<0.001
Duration of disease (years)	3.2±4.6	7.5±7.4	12.5±8.7	<0.001
Chlorpromazine-equivalent dose	454.7±181.2	396.8±188.3	281.7±135.6	<0.001
Number of episodes				<0.001
FE	26 (60.5%)	61 (26.8%)	2 (3.6%)	
2-3 episodes	11 (25.6%)	84 (36.8%)	15 (26.8%)	
4-6 episodes	4 (9.3%)	54 (23.7%)	22 (39.3%)	
Over 6 episodes	2 (4.7%)	29 (12.7%)	17 (30.4%)	

A. the Second Xiangya Hospital of Central South University; B. the Second People's Hospital of Hunan Province; C. Changsha Psychiatric Hospital.

Supplementary Table 3 Changes in membrane fatty acid levels of patient subgroups after antipsychotic treatment.

Fatty acids	FE					2-3 episodes					4-6 episodes					Over 6 episodes				
	SZ-0w	SZ-4w	Z	p		SZ-0w	SZ-4w	Z	p		SZ-0w	SZ-4w	Z	p		SZ-0w	SZ-4w	Z	p	
C16:0	472.85±22.09	652.3±32.11	-7.38	<0.001	↑	485.04±19.62	597.96±24.49	-6.041	<0.001	↑	593.09±32.16	539.73±29.89	-2.168	0.030	↓	611.40±45.52	473.85±32.18	-3.908	<0.001	↓
C18:0	244.7±12.14	341.14±16.43	-7.64	<0.001	↑	242.18±9.95	286.25±11.67	-4.943	<0.001	↑	285.51±14.9	260.32±14.38	-2.628	0.009	↓	297.92±19.8	237.97±17.82	-3.313	<0.001	↓
C18:1n9c	289.54±12.83	429.66±22.47	-7.51	<0.001	↑	297.45±13.16	360.81±15.45	-5.259	<0.001	↑	369.03±22.25	338.93±19.44	-1.573	0.116	ns	371.03±28.53	277.61±22.64	-3.846	<0.001	↓
C18:2n6c	288.67±15.14	458.88±25.94	-7.12	<0.001	↑	302.28±15.12	382.69±17.51	-4.854	<0.001	↑	390.59±22.4	373.07±20.55	-0.321	0.748	ns	390.12±38.51	317.28±29.48	-2.585	0.010	↓
C20:3n6	10.42±0.65	19.99±1.28	-7.58	<0.001	↑	12.04±0.85	15.11±0.99	-4.138	<0.001	↑	15.16±1.29	15.15±1.14	-0.377	0.707	ns	16.35±1.76	12.98±1.42	-2.831	0.005	↓
C20:4n6	170.17±8.44	246.43±12.17	-7.42	<0.001	↑	179.47±8.52	209.55±9.02	-3.814	<0.001	↑	239.66±13.78	188.33±10.14	-5.079	<0.001	↓	231.84±18.36	172.77±14.83	-4.400	<0.001	↓
C20:5n3	2.59±0.34	6.45±0.56	-7.98	<0.001	↑	2.65±0.18	3.36±0.28	-3.434	<0.001	↑	4.94±0.76	3.27±0.37	-4.048	<0.001	↓	4.41±0.40	2.95±0.33	-4.677	<0.001	↓
C22:4n6	25.42±1.41	41.04±1.99	-7.96	<0.001	↑	27.43±1.57	30.81±1.53	-3.276	0.001	↑	37.51±2.65	28.69±1.93	-5.621	<0.001	↓	38.25±3.20	27.45±2.81	-4.041	<0.001	↓
C22:5n3	18.53±1.54	31.17±2.17	-7.99	<0.001	↑	20.42±1.52	24.34±2.03	-4.213	<0.001	↑	26.96±2.95	20.81±2.01	-4.441	<0.001	↓	24.12±2.69	17.81±1.93	-4.339	<0.001	↓
C22:6n3	29.52±1.65	48.70±2.12	-8.03	<0.001	↑	32.73±1.59	37.39±1.75	-3.440	<0.001	↑	45.34±3.49	33.09±2.45	-5.756	<0.001	↓	42.26±3.81	29.63±2.93	-4.810	<0.001	↓

Mean±SEM; FE, first episode; Wilcoxon Signed Ranks Test.

Supplementary Table 4 Clinical characteristics of patients in more-responsive and less-responsive subgroups

Characteristics	More-responsive				Less-responsive			
	0-5 yr(s)	5-10 yrs	>10 yrs	Total	0-5 yr(s)	5-10 yrs	>10 yrs	Total
N	141	37	21	199	18	45	65	128
Male (%)	69 (48.9%)	15 (40.5%)	7(33.3%)	91 (45.7%)	9 (50.0%)	17(37.8%)	30 (46.2%)	56 (43.8%)
Age (years)	28.49±9.68	29.59±7.29	42.38±8.58	30.16±10.06	28.89±7.71	32.87±6.88	41.43±9.39	36.66±9.70
Range	15-59	20-53	29-57	15-59	16-46	19-49	18-57	16-57
BMI (kg/m2)	20.88±3.02	22.10±4.12	21.61±3.20	21.20±3.30	22.02±3.74	24.09±4.87	23.10±4.25	23.29±4.43
Duration of disease (years)	1.84±1.58	8.14±1.50	18.39±8.07	4.76±6.06	3.70±1.34	7.76±1.31	18.26±6.79	12.52±7.75
Chlorpromazine-equivalent dose	395.0±179.7	423.4±187.6	420.1±219.4	403.1±185.0	369.1±251.5	344.7±151.8	360.0±197.7	356.2±184.9

Values represent as mean ± standard deviation; More responsive subgroup consists of patients on 1-3 episode(s) and less-responsive subgroup consists of patients on over 3 episodes. BMI, body mass index; yr(s), year(s).

Supplementary Table 5 Demographic characteristics of male and female patients

	Male	Female	p
n	147	180	
Age (years)	30.89±10.02	34.18±10.50	**0.004**
BMI (kg/m2)	22.40 ±3.47	21.61 ±3.84	0.066
Duration of disease (years)	7.24 ±6.89	8.26 ±8.37	0.237
Chlorpromazine-equivalent dose	406.55 ±190.93	366.88 ±180.25	0.062
Number of episodes			0.366
FE	44 (29.9%)	45 (25.0%)	
2-3 episodes	47 (32.0%)	63 (35.0%)	
4-6 episodes	31 (21.1%)	49 (27.2%)	
Over 6 episodes	25 (17.0%)	23 (12.8%)	

Supplementary Table 6 Meal composition for patients

Meal	Composition	Caloric Value (kcal)	Protein (g)	Fat (g)	Carbohydrate (g)
Breakfast	Egg 50g, meat bun 100g, steamed roll 90g, rice porridge 250g	608	22.7	16.2	97.4
Lunch	Rice 200g, vegetables 250g, meat 200g， oil 33g	806	48	36	76.2
Dinner	Rice 200g, vegetables 375g, meat 75g， oil 18g	611	24.8	22.5	81.6

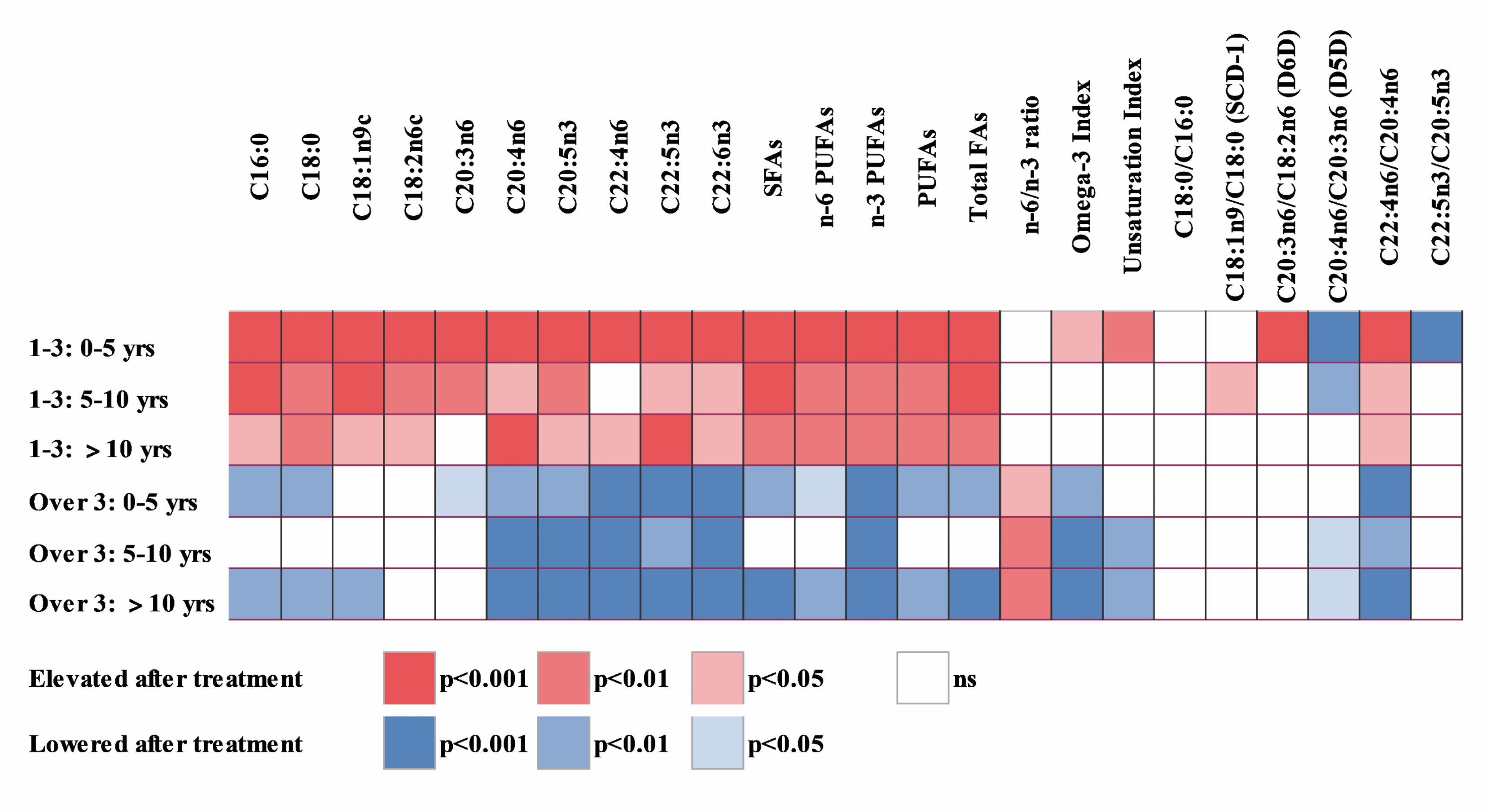

Supplementary Figure 1 Changes in membrane fatty acid levels and ratios of patients with different durations of illness disease in more-responsive (1-3 episodes) and less-responsive (over 3 episodes) subgroups after antipsychotic treatment. ns, not significant; yrs, years.

		Δ C16:0	Δ C18:0	Δ C18:1n9c	Δ C18:2n6c	Δ C20:3n6	Δ C20:4n6	Δ C20:5n3	Δ C22:4n6	Δ C22:5n3	Δ C22:6n3	Δ SFAs	Δ n-6 PUFAs	Δ n-3 PUFAs	Δ PUFAs	Δ Total FAs	Δ n-6/n-3 ratio	Δ Omega-3 Index	Δ UI	Δ C18:0/C16:0	Δ C18:1n9/C18:0	Δ C20:3n6/C18:2n6	Δ C20:4n6/C20:3n6	Δ C22:4n6/C20:4n6	Δ C22:5n3/C20:5n3
	Positive syndrome	0.063	0.051	0.089	0.053	0.090	0.080	0.120	0.035	0.024	0.070	0.062	0.069	0.064	0.070	0.075	-0.045	0.018	0.051	0.012	0.056	0.004	0.029	0.047	-0.012
	Negative syndrome	0.111	0.081	0.127	0.073	0.137	0.116	0.145	0.103	0.118	0.097	0.107	0.100	0.119	0.105	0.117	-0.037	0.004	0.006	-0.044	0.050	0.049	-0.010	0.085	-0.053
	General psychopathology	0.142	0.123	0.164	0.138	0.135	0.117	0.142	0.114	0.123	0.113	0.143	0.145	0.130	0.147	0.158	-0.019	-0.012	0.034	-0.033	0.086	-0.010	0.030	0.123	-0.089
	Anergia	0.104	0.110	0.129	0.060	0.104	0.130	0.126	0.111	0.124	0.114	0.111	0.095	0.129	0.102	0.118	-0.049	0.045	0.020	-0.003	0.017	0.034	-0.017	0.097	-0.042
Total	Thought disturbance	0.066	0.070	0.079	0.039	0.067	0.055	0.081	0.024	-0.032	0.054	0.071	0.049	0.029	0.048	0.067	0.004	-0.020	0.023	0.047	0.047	-0.001	0.047	0.039	-0.015
	Activation	0.093	0.066	0.139	0.091	0.029	0.081	0.111	0.021	0.081	0.116	0.089	0.092	0.112	0.097	0.108	-0.038	0.060	0.031	-0.032	0.063	-0.081	0.074	-0.001	-0.065
	Paranoid belligerence	0.074	0.039	0.101	0.072	0.069	0.083	0.125	0.066	0.066	0.072	0.066	0.084	0.082	0.086	0.086	-0.033	0.034	0.061	-0.051	0.096	-0.025	0.073	0.052	-0.022
	Depression	0.094	0.127	0.150	0.138	0.085	0.121	0.114	0.088	0.127	0.106	0.110	0.143	0.124	0.145	0.140	0.024	-0.033	0.016	0.028	0.033	-0.075	0.048	0.019	-0.121
	PANSS total score	0.145	0.105	0.147	0.118	0.148	0.122	0.159	0.109	0.087	0.122	0.139	0.133	0.123	0.135	0.147	-0.033	0.013	0.024	-0.050	0.076	0.117	0.029	0.124	-0.061
	Positive syndrome	-0.078	0.045	-0.031	-0.121	-0.079	0.005	0.071	-0.155	-0.163	-0.030	-0.044	-0.104	-0.087	-0.108	-0.072	-0.034	-0.006	-0.051	0.063	-0.082	0.023	-0.044	-0.023	0.023
	Negative syndrome	0.040	-0.023	0.116	-0.024	0.157	0.047	0.190	0.001	0.069	0.105	0.023	0.001	0.125	0.012	0.045	-0.089	0.088	-0.029	-0.170	0.111	0.230	-0.081	0.010	-0.037
	General psychopathology	-0.026	-0.032	-0.001	-0.087	0.038	-0.134	0.091	-0.159	-0.036	-0.067	-0.030	-0.114	-0.040	-0.114	-0.061	0.000	-0.037	-0.199	-0.140	0.011	0.113	-0.073	-0.030	-0.124
	Anergia	0.021	0.006	0.076	-0.082	0.108	0.024	0.177	-0.008	0.040	0.155	0.018	-0.053	0.135	-0.040	0.011	-0.173	0.153	-0.067	-0.091	0.032	0.225	-0.092	-0.011	-0.049
FE	Thought disturbance	-0.024	0.092	-0.012	-0.104	-0.067	-0.077	0.123	-0.238	-0.274	-0.033	0.012	-0.118	-0.136	-0.126	-0.051	0.040	-0.001	-0.098	0.101	-0.069	0.008	-0.003	-0.072	-0.011
	Activation	-0.114	-0.075	-0.057	-0.145	-0.081	-0.116	0.135	-0.112	-0.008	-0.029	-0.111	-0.157	0.003	-0.151	-0.124	-0.082	0.097	-0.045	0.030	-0.044	0.059	0.062	0.021	-0.121
	Paranoid belligerence	-0.056	0.014	-0.015	-0.118	-0.052	0.025	0.089	-0.047	-0.009	-0.009	-0.038	-0.090	0.006	-0.086	-0.056	-0.019	-0.008	-0.076	-0.029	-0.028	0.005	0.024	-0.019	0.012
	Depression	0.040	0.032	0.157	0.052	0.166	-0.016	0.143	0.022	0.162	0.016	0.041	0.044	0.116	0.052	0.080	0.072	-0.033	-0.124	-0.094	0.096	0.102	-0.070	-0.019	-0.215
	PANSS total score	-0.025	-0.006	0.017	-0.103	0.033	-0.038	0.127	-0.118	-0.046	-0.001	-0.021	-0.096	-0.002	-0.093	-0.043	-0.040	0.012	-0.130	-0.105	0.002	0.122	-0.062	-0.020	-0.063
	Positive syndrome	0.086	0.056	0.133	0.025	0.147	0.083	0.278	-0.013	-0.024	0.092	0.081	0.053	0.072	0.056	0.091	-0.003	-0.009	-0.019	-0.017	0.146	-0.036	0.044	-0.075	-0.218
	Negative syndrome	-0.010	-0.036	-0.019	-0.068	-0.005	0.025	-0.008	-0.044	-0.014	-0.006	-0.019	-0.041	-0.010	-0.039	-0.030	-0.032	-0.048	-0.105	-0.013	-0.029	0.008	-0.084	-0.062	-0.119
	General psychopathology	0.224	0.202	0.189	0.126	0.115	0.163	0.105	0.094	0.012	0.125	0.231	0.156	0.093	0.154	0.213	0.041	-0.102	-0.061	-0.018	0.004	-0.066	0.138	0.015	-0.064
	Anergia	-0.052	-0.029	0.012	-0.046	0.000	0.096	0.004	0.019	0.095	0.043	-0.048	0.004	0.069	0.010	-0.014	0.008	-0.027	0.026	0.000	0.017	0.003	0.010	0.024	-0.050
2-3	Thought disturbance	0.123	0.112	0.152	0.027	0.164	0.117	0.172	0.051	-0.037	0.136	0.127	0.070	0.083	0.074	0.123	-0.007	-0.011	-0.004	0.024	0.114	-0.022	0.072	-0.032	-0.169
	Activation	0.172	0.081	0.168	0.089	0.020	0.074	0.112	-0.106	0.009	0.126	0.154	0.084	0.093	0.087	0.144	-0.019	-0.018	-0.086	-0.108	0.134	-0.145	0.116	-0.126	-0.053
	Paranoid belligerence	0.093	0.064	0.126	0.032	0.061	0.071	0.201	0.011	-0.036	0.046	0.089	0.051	0.030	0.050	0.090	-0.002	-0.056	-0.053	-0.032	0.104	-0.080	0.092	-0.071	-0.215
	Depression	0.080	0.098	0.099	0.064	-0.028	0.138	-0.038	-0.006	-0.029	0.020	0.090	0.093	-0.003	0.086	0.100	0.067	-0.122	-0.035	0.042	-0.025	-0.135	0.175	-0.110	0.043
	PANSS total score	0.157	0.124	0.151	0.067	0.113	0.119	0.157	0.032	-0.015	0.106	0.156	0.095	0.074	0.096	0.145	0.009	-0.077	-0.078	-0.020	0.045	-0.044	0.071	-0.021	-0.156
	Positive syndrome	-0.014	-0.087	-0.010	0.012	-0.029	-0.147	-0.116	-0.231	-0.200	-0.218	-0.040	-0.053	-0.217	-0.074	-0.049	0.161	-0.405	-0.197	0.017	0.117	0.095	0.133	-0.023	0.034
	Negative syndrome	0.092	0.066	0.056	0.055	0.028	0.025	0.040	-0.026	0.030	-0.045	0.086	0.046	-0.013	0.040	0.065	0.064	-0.244	-0.043	-0.019	-0.002	0.071	0.196	0.064	-0.111
	General psychopathology	-0.027	-0.047	0.065	0.096	-0.135	-0.082	-0.047	-0.193	-0.057	-0.186	-0.035	0.026	-0.140	0.006	0.001	0.192	-0.405	-0.042	0.020	0.173	-0.129	0.141	-0.054	0.011
	Anergia	0.122	0.122	0.082	0.048	-0.057	0.056	0.038	0.007	-0.004	-0.011	0.127	0.050	-0.004	0.045	0.090	0.016	-0.101	-0.040	0.038	-0.060	-0.024	0.141	-0.002	0.054
4-6	Thought disturbance	0.032	-0.004	0.076	0.094	-0.021	-0.111	-0.122	-0.175	-0.203	-0.188	0.021	0.019	-0.200	-0.008	0.023	0.256	-0.422	-0.193	0.085	0.152	0.012	0.110	0.025	0.046
	Activation	0.130	0.114	0.228	0.253	0.022	0.081	-0.020	-0.052	-0.013	0.000	0.129	0.199	-0.007	0.178	0.176	0.242	-0.302	-0.066	0.013	0.191	-0.079	0.116	-0.128	-0.005
	Paranoid belligerence	-0.013	-0.126	0.004	0.057	-0.083	-0.122	-0.062	-0.227	-0.137	-0.200	-0.053	-0.015	-0.178	-0.036	-0.036	0.193	-0.332	-0.096	-0.108	0.178	-0.003	0.192	-0.072	-0.056
	Depression	0.174	0.197	0.193	0.309	0.025	0.166	0.169	0.047	0.151	0.153	0.189	0.271	0.167	0.264	0.228	0.029	-0.086	0.088	0.039	0.005	-0.243	0.165	0.025	-0.076
	PANSS total score	0.028	-0.035	-0.007	0.057	-0.070	-0.112	-0.051	-0.184	-0.173	-0.174	0.007	-0.009	-0.173	-0.029	-0.010	0.164	-0.372	-0.138	-0.020	0.043	-0.007	0.184	-0.004	-0.012
	Positive syndrome	-0.079	-0.090	-0.056	0.066	0.023	0.092	0.090	0.169	0.280	0.238	-0.092	0.090	0.263	0.108	-0.009	-0.182	0.226	0.293	-0.064	0.045	0.019	-0.347	0.104	0.104
	Negative syndrome	0.153	0.092	0.185	0.176	0.109	0.212	0.166	0.268	0.178	0.112	0.145	0.219	0.147	0.216	0.195	0.282	-0.253	0.106	0.068	0.104	-0.326	0.012	0.302	0.066
	General psychopathology	0.114	0.064	0.103	0.170	0.169	0.150	0.062	0.284	0.249	0.230	0.106	0.195	0.245	0.203	0.154	0.002	-0.077	0.174	0.014	0.190	-0.088	-0.121	0.334	0.068
	Anergia	0.067	0.144	0.079	0.051	0.098	0.181	0.088	0.268	0.165	0.122	0.106	0.121	0.144	0.125	0.116	0.147	-0.105	-0.003	0.198	-0.057	-0.098	-0.124	0.309	0.030
Over 6	Thought disturbance	-0.052	-0.031	-0.123	-0.019	0.064	0.088	0.172	0.240	0.349	0.211	-0.049	0.036	0.271	0.058	-0.025	-0.256	0.111	0.226	-0.038	0.048	0.087	-0.252	0.164	0.190
	Activation	-0.225	-0.191	-0.112	-0.169	-0.189	-0.024	-0.066	0.041	0.098	0.099	-0.236	-0.128	0.096	-0.111	-0.176	-0.174	0.299	0.252	-0.081	0.035	0.101	-0.320	0.183	0.024
	Paranoid belligerence	-0.061	-0.126	-0.056	0.067	-0.010	0.057	-0.095	0.160	0.152	0.225	-0.095	0.077	0.201	0.090	-0.018	-0.129	0.312	0.347	-0.103	0.148	-0.059	-0.234	0.083	0.077
	Depression	0.034	0.206	0.105	0.021	-0.005	0.090	-0.111	0.151	0.084	0.092	0.108	0.057	0.084	0.060	0.096	0.079	-0.257	-0.087	0.263	0.049	-0.139	0.020	0.246	0.125
	PANSS total score	0.161	-0.006	0.144	0.255	0.244	0.221	0.190	0.354	0.374	0.309	0.110	0.286	0.349	0.297	0.204	0.039	0.037	0.221	-0.130	0.166	-0.189	-0.211	0.232	0.116

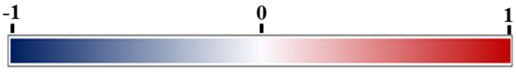

Supplementary Figure 2 Heatmap of correlation coefficients between fatty acid level and ratio changes and symptom improvement in schizophrenia patients after antipsychotic treatment.
