# Supplementary material for: Reduced erythrocyte membrane polyunsaturated fatty acid levels indicate diminished treatment response in patients with multi- versus first-episode schizophrenia
Source: Schizophrenia (Heidelb). 2022 Feb 25;8(1):7. doi: 10.1038/s41537-022-00214-2 (PMC8881498; doi:10.1038/s41537-022-00214-2)
Supplement: Supplementary file 2 — Reporting Summary [file 41537_2022_214_MOESM2_ESM.pdf]

nature portfolio

Corresponding author(s): Hualin Cai

Last updated by author(s): Jan 28, 2022

Reporting Summary

Nature Portfolio wishes to improve the reproducibility of the work that we publish. This form provides structure for consistency and transparency in reporting. For further information on Nature Portfolio policies, see our Editorial Policies and the Editorial Policy Checklist.

Statistics

For all statistical analyses, confirm that the following items are present in the figure legend, table legend, main text, or Methods section.

n/a	Confirmed	
☐	☒	The exact sample size (n) for each experimental group/condition, given as a discrete number and unit of measurement
☐	☒	A statement on whether measurements were taken from distinct samples or whether the same sample was measured repeatedly
☐	☒	The statistical test(s) used AND whether they are one- or two-sided<br>*Only common tests should be described solely by name; describe more complex techniques in the Methods section.*
☐	☒	A description of all covariates tested
☐	☒	A description of any assumptions or corrections, such as tests of normality and adjustment for multiple comparisons
☐	☒	A full description of the statistical parameters including central tendency (e.g. means) or other basic estimates (e.g. regression coefficient) AND variation (e.g. standard deviation) or associated estimates of uncertainty (e.g. confidence intervals)
☐	☒	For null hypothesis testing, the test statistic (e.g. F, t, r) with confidence intervals, effect sizes, degrees of freedom and P value noted<br>*Give P values as exact values whenever suitable.*
☒	☐	For Bayesian analysis, information on the choice of priors and Markov chain Monte Carlo settings
☒	☐	For hierarchical and complex designs, identification of the appropriate level for tests and full reporting of outcomes
☐	☒	Estimates of effect sizes (e.g. Cohen's d, Pearson's r), indicating how they were calculated

Our web collection on statistics for biologists contains articles on many of the points above.

Software and code

Policy information about availability of computer code

Data collection	n/a
Data analysis	The data analyses were performed using SPSS version 18.0 software.

For manuscripts utilizing custom algorithms or software that are central to the research but not yet described in published literature, software must be made available to editors and reviewers. We strongly encourage code deposition in a community repository (e.g. GitHub). See the Nature Portfolio guidelines for submitting code & software for further information.

Data

Policy information about availability of data

All manuscripts must include a data availability statement. This statement should provide the following information, where applicable:

- Accession codes, unique identifiers, or web links for publicly available datasets
- A description of any restrictions on data availability
- For clinical datasets or third party data, please ensure that the statement adheres to our policy

The data that support the findings of this study are available from the corresponding author upon reasonable request.

Field-specific reporting

Please select the one below that is the best fit for your research. If you are not sure, read the appropriate sections before making your selection.

☒ Life sciences ☐ Behavioural & social sciences ☐ Ecological, evolutionary & environmental sciences

For a reference copy of the document with all sections, see nature.com/documents/nr-reporting-summary-flat.pdf

Life sciences study design

All studies must disclose on these points even when the disclosure is negative.

Sample size	This is an exploratory research, so we recruited as many participants as possible during the research period.
Data exclusions	Participants who dropped out during the four-week follow-up were excluded in the analysis. The blood sample collection and/or PANSS evaluation after four-week treatment were not performed because they were discharged from hospital in advance. We also excluded patients who were diagnosed as bipolar disorder etc. after follow up.
Replication	To verify the repeatability of the fatty acid analysis, each blood sample was tested in triplicates and averaged for the final result.
Randomization	This is an observational study on schizophrenia patients and healthy controls. Random allocation was not involved in the research.
Blinding	This is basically an observational study on schizophrenia patients and healthy controls. We did not intervene in the treatment process or the therapeutic strategy for patients. Blinding was not applicable in the research.

Reporting for specific materials, systems and methods

We require information from authors about some types of materials, experimental systems and methods used in many studies. Here, indicate whether each material, system or method listed is relevant to your study. If you are not sure if a list item applies to your research, read the appropriate section before selecting a response.

Materials & experimental systems

n/a	Involved in the study	
☒	☐	Antibodies
☒	☐	Eukaryotic cell lines
☒	☐	Palaeontology and archaeology
☒	☐	Animals and other organisms
☐	☒	Human research participants
☐	☒	Clinical data
☒	☐	Dual use research of concern

Methods

n/a	Involved in the study	
☒	☐	ChIP-seq
☒	☐	Flow cytometry
☒	☐	MRI-based neuroimaging

Human research participants

Policy information about studies involving human research participants

Population characteristics	A cohort of 327 patients with schizophrenia and 159 healthy controls from 2016-2018 was recruited and the characteristics of the subjects are presented in Supplementary Table 1. There were no significant differences in sex or age between these two groups of participants. Patients were divided into four subgroups according to the times of relapse at enrollment, and their characteristics are also shown in Supplementary Table 1. Nearly half of the patients were treated with risperidone or olanzapine, while 30% of the patients received combination treatment with AAPDs. The chlorpromazine-equivalent dose was calculated using the defined daily dose (DDD) method. Patients with 2-3 episodes received the largest doses, followed by the first-episode subgroup, although the difference was not statistically significant.
Recruitment	The patients were recruited in the order of admission. Patients aged 15-60 years were recruited at three centers. All participants were Han Chinese who met the following inclusion criteria: (1) DSM-5 criteria for schizophrenia, schizoaffective disorder, or schizophreniform disorder; and (2) newly hospitalized with first-onset, or recurrent psychosis and without taking antipsychotic drugs for more than one month prior to admission. We excluded patients complicated with Alzheimer's disease, epilepsy, alcohol or drug abuse, or other serious physical or mental disorders. Patients who were taking medicine for hyperlipidemia or diabetes mellitus were also excluded. The healthy controls who matched the patients in age and sex were recruited from the same area.
Ethics oversight	The experimental protocol was approved by the Ethics Committee of the Second Xiangya Hospital of Central South University, the Second People's Hospital of Hunan Province and Changsha Psychiatric Hospital.

Note that full information on the approval of the study protocol must also be provided in the manuscript.

Clinical data

Policy information about clinical studies

All manuscripts should comply with the ICMJE guidelines for publication of clinical research and a completed CONSORT checklist must be included with all submissions.

Clinical trial registration	The experimental protocol was registered in the Chinese Clinical Trials Registry (http://www.chictr.org.cn/) as ChiCTR-OOC-16008988.
Study protocol	The study protocol is available from the corresponding author upon reasonable request.
Data collection	The patients were recruited at the Second Xiangya Hospital of Central South University, the Second People's Hospital of Hunan Province and Changsha Psychiatric Hospital.
Outcomes	At enrollment and after 4 weeks of treatment, patients were rated on the Positive and Negative Syndrome Scale (PANSS) performed by three senior experienced psychiatrists. The PANSS total score, three scales (positive syndrome, negative syndrome and general psychopathology) and five cluster scores (anergia, thought disturbance, activation, paranoid belligerence, depression) were used to assess the psychopathological states.<br>Fasting blood samples were collected in the morning (7 am) at baseline and after 4 weeks of antipsychotic treatment for patients. Healthy controls were examined only once. Erythrocyte membrane fatty acid levels were analyzed.
